# Perfecting Sensory Restoration and the Unmet Need for Personalized Medicine in Cochlear Implant Users: A Narrative Review

**DOI:** 10.3390/brainsci15050479

**Published:** 2025-05-01

**Authors:** Archana Podury, Brooke Barry, Karen C. Barrett, Nicole T. Jiam

**Affiliations:** 1 Department of Head and Neck Surgery, University of California, San Diego, CA 92093, USA; apodury@health.ucsd.edu; 2 Department of Head and Neck Surgery, University of California, San Francisco, CA 94143, USA; brooke.barry@ucsf.edu (B.B.); karen.barrett@ucsf.edu (K.C.B.); 3 Institute for Health and Aging, University of California, San Francisco, CA 94143, USA

**Keywords:** hearing loss, cochlear implant, music, pitch perception, precision medicine

## Abstract

Hearing loss is one of the most common and undertreated medical conditions worldwide, with an estimated 466 million people (5% of the world’s population) reporting disabling hearing impairment. The implications are significant; untreated hearing loss increases the risk of depression, social isolation, unemployment, cognitive decline, and falls. Cochlear implants (CIs) are surgically implanted electrical devices that allow people with severe hearing loss to process sound. Over the past 50 years, CI development has made remarkable ground, such that most CI users have adequate speech perception in a silent environment. These language achievements, while significant milestones, fall short of perfect sensory restoration. Many of these limitations with complex sound perception are due to our one-size-fits-all approach towards CIs and speech-based metrics for evaluating implant performance. In the past decade, there has been exponential interest in improving CI-mediated music perception, as it serves as a key conduit to restoring normal hearing. The present literature demonstrates the need for a personalized approach towards cochlear implantation and management. Our proposed narrative review illustrates the limitations of CI-mediated sound processing and discusses ways in which precision medicine can be introduced into the ever-expanding hearing loss population.

## 1. Introduction

Hearing is the primary afferent system of social life [1]. Throughout history, hearing has served as the foundation of language, emotional connection, and community [2]. Sound allows us to navigate both our environment and our relationships. Among the many forms of sound, music stands as the most intricate and expressive, as it integrates complex acoustic features to convey meaning beyond spoken language. By allowing us to interact with these forms of expression, hearing is fundamental to identity and quality of life [3,4,5].

Hearing loss is one of the most prevalent and undertreated medical conditions [6]. An estimated 466 million people, or 5% of the world’s population, report disabling hearing impairment, and this number is expected to increase to 900 million by 2050 as our population continues to age [7]. Untreated hearing loss is associated with significant morbidity, including depression, cognitive decline, and falls [8,9,10]. Hearing loss is also associated with numerous psychosocial impacts, including social isolation, unemployment, and increased reliance on public support services [11,12]. As a result, hearing loss is considered a modifiable risk factor for various complex comorbidities, and there is increased interest in understanding the mechanisms of and developing nuanced therapeutics for hearing loss [13].

Mechanisms of hearing loss are multifold. Sound perception involves the conduction of sound waves to the outer ear, the bony middle ear, the sensorineural apparatus of the inner ear, and, via the auditory nerve, to higher-level auditory processing centers, and hearing loss may occur at any point during this pathway [14,15]. Conductive hearing loss is defined as pathologies in the outer or middle ear that prevent transmission of sound to the inner ear. Sensorineural hearing loss is defined as pathologies of the inner ear or auditory nerve that disrupt sound perception. Patients may also experience mixed hearing loss or a combination of both mechanisms. The severity of hearing loss is classified as mild, moderate, severe, or profound, depending on the intensity of sound required to detect pure tones at various pitches. This classification system has helped broadly stratify patients for appropriate treatment approaches [16].

Over the past 50 years, cochlear implants (CIs) have emerged as highly successful neural prostheses that can restore speech perception in patients with significant unilateral or bilateral sensorineural hearing loss [17]. CIs bypass dysfunctional hair cells in the cochlea and directly stimulate the auditory nerve via an implantable electrode array. CI users demonstrate robust speech outcomes, with many patients reporting significant improvements in communication and quality of life [18,19].

While speech outcomes are excellent, there remain significant limitations in CI performance for more structurally complex sounds, such as speech perception in noisy environments and music perception [20,21]. Much of this discrepancy in outcomes stems from the technical limitations in present-day CI technologies as well as a longstanding emphasis in the field on optimizing speech outcomes over complete sensory restoration. Ref. [22] This review discusses our current understanding of sensory transduction and the limitations of CI-mediated sound processing and examines how integrating personalized medicine into cochlear implantation and targeting music perception as a gold-standard outcome may allow for advances beyond speech comprehension and towards complete sensory restoration.

## 2. Methods

This article discusses the relevant literature in the form of a narrative review. A thorough and focused literature review was conducted on the following subtopics pre-determined by the authors: (1) sensory transduction of sound; (2) CI design; (3) music perception in CI users; and (4) personalized medicine approaches in CI. Pubmed, Web of Science, and Embase were searched for articles, published between 1970 and 2025, that were relevant to each subtopic. MeSH search terms included “cochlear implant”, “cochlear implantation”, “auditory perception”, “pitch”, “timbre”, “music”, “music perception”, “music enjoyment”, “music experience”, “personalized medicine”, and “precision medicine”. Studies were reviewed for relevance to each subtopic, methodology, and appropriate assessment of results. References from select articles were hand-searched for additional relevant studies. Exclusion criteria were a lack of relevance to the subtopic, redundant material, non-English articles, and abstracts without full-text availability. The findings from the key articles are summarized in a narrative format.

## 3. Sound Perception

**Figure 1:**
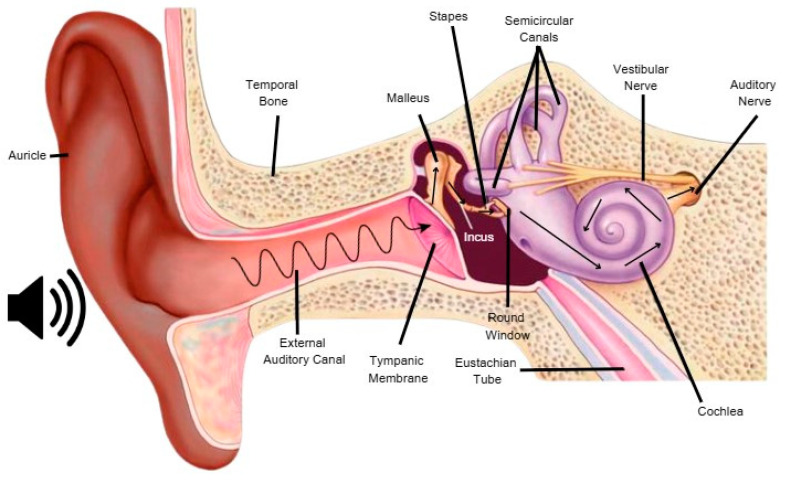
The transduction of sound is illustrated with black arrows. Sound waves are guided into the external auditory canal by the auricle. These waves hit the tympanic membrane, which vibrates the three ear bones: malleus, incus, and stapes. The footplate of the stapes vibrates the round window, which stimulates the movement of a fluid wave in the cochlea. This fluid movement triggers the movement of hair cells, which stimulates the production of an action potential in the auditory nerve. This image was adapted from https://doi.org/10.5772/intechopen.105466, accessed on 1 March 2025.

Sound is a pressure transduction wave that propagates via rhythmic compressions and rarefactions of air molecules. The sensory transduction pathway begins in the outer ear, where sound waves are directed by the auricle and external auditory canal to the tympanic membrane. The tympanic membrane transmits vibrations to the ossicular chain in the middle ear. The ossicular chain consists of three mobile bones, the malleus, incus, and stapes, which function as a lever system and amplify sound. The stapes footplate transmits the vibrational signal to the inner ear via the oval window, which generates a fluid wave within the cochlea. This fluid wave travels towards the round window and generates vibrations across the length of the cochlear basilar membrane. This process initiates the conversion of mechanical energy to electrical information transmitted to the brain (Figure 1) [23,24].

The cochlea is spiral-shaped and coils around a central axis, known as the modiolus, which contains the auditory nerve and associated spiral ganglion cells. The cochlea is divided into three fluid-filled chambers: the scala vestibuli, scala media, and scala tympani. The scala vestibuli and tympani are filled with perilymph and facilitate the transmission of the cochlear fluid wave. The scala media contains potassium-rich endolymph and houses the Organ of Corti. This hearing organ consists of hair cells that rest on a mobile basilar membrane and are fixed to a tectorial membrane via stereocilia tip-links. Vibrations in the basilar membrane bend the tip-links and open mechanically gated potassium channels. The surrounding potassium-rich endolymph drives depolarization of the hair cells. This signal is transmitted along the afferent hearing pathway, from the auditory nerve fibers to the spiral ganglion cells, the cochlear division of the eighth cranial nerve, and the brainstem cochlear nuclei. The brainstem projects to the superior olivary nuclei, lateral lemniscus, and inferior colliculus for further processing. The auditory input continues to travel to the medial geniculate nucleus of the thalamus, primary auditory cortex, and higher-order associative cortices [25].

The auditory system must not only detect sound but must also accurately represent essential acoustic elements, such as loudness, pitch, and timbre. Loudness is defined as the perceived intensity of sound. Loudness is thought to be related to the degree of displacement of the basilar membrane and modulation by outer hair cells, which is ultimately encoded as an action potential frequency within the auditory nerve [26,27]. Pitch is defined as the perceived frequency of sound and is often thought of as the primary auditory sensation [28]. Timbre is defined as the perceptual quality that distinguishes between sounds of the same pitch and loudness, often described as tone color. Accurate timbre perception relies on the high-fidelity transmission of rapidly fluctuating spectral–temporal information about overlapping harmonics, decay, and resonance [29].

**Figure 2:**
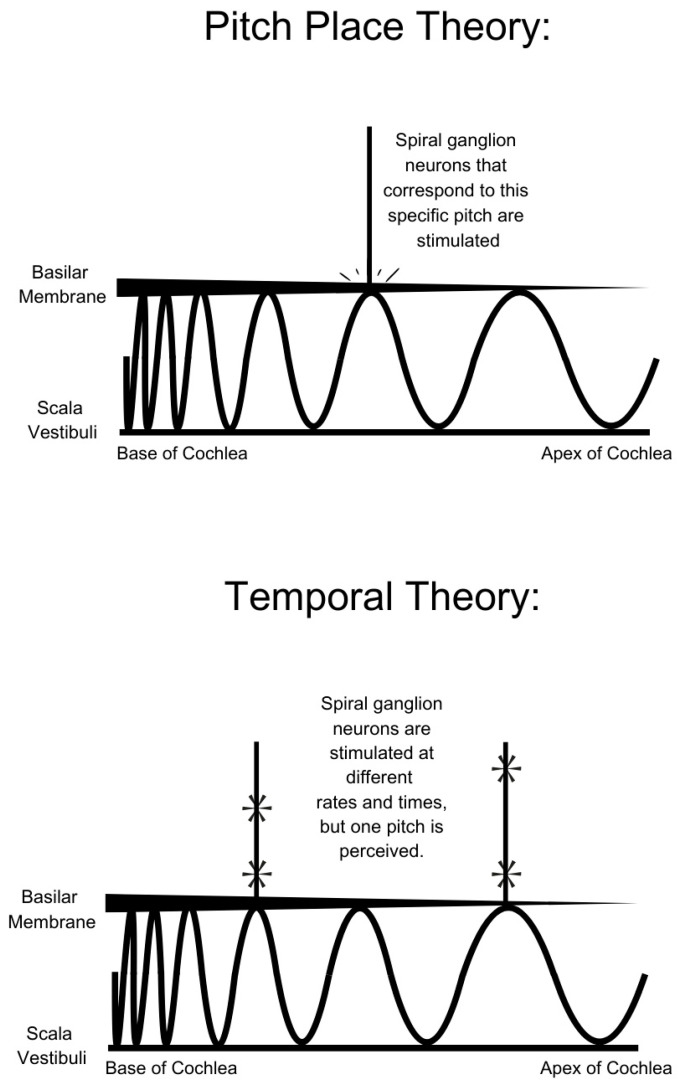
In the Pitch–Place Theory, the fluid wave within the scala vestibuli stimulates the basilar membrane most at a unique place in the cochlea, dependent on the frequency of the wave. This input is then sent through the spiral ganglion neurons to the auditory nerve and then to the auditory cortex. In Temporal Pitch Theory, the fluid wave is perceived in multiple different spiral ganglion neurons. The frequency of the pitch is determined by the rate of action potentials traveling up the spiral ganglion neurons. According to the Volley Theory, the frequency of the perceived pitch can be higher than the rate of action potentials in each individual spiral ganglion neuron. This is because the action potentials are produced at different times in relation to one another and can additively indicate each start of a period of the sinusoidal curve that represents the perceived sound wave in the auditory nerve.

The perception of pitch is explained by two primary theories (Figure 2). The Pitch–Place Theory states that pitch perception is determined by the location of maximal vibration along the basilar membrane in response to a given frequency of sound [30]. The basilar membrane is thickest at the base and thinnest at the apex. High frequencies resonate along the base, while low frequencies resonate along the apex. This property lends to tonotopic organization of the cochlea, where frequency information is encoded by the location of activated hair cells along the basilar membrane. Tonotopic organization is mathematically described by the Greenwood function, which provides a logarithmic relationship between cochlear place and perceived pitch and is frequently used in CI programming [31,32]:

f = A × (10^ax^ – k), (1)

where f is the frequency of sound; x is the fractional length along the basilar membrane, measured from the apex; and A, k, and a are scaling constants based on the average human cochlear dimensions.

The Temporal Pitch Theory states that pitch is encoded by the timing of action potentials within the auditory nerve. By Fourier’s theorem, all sound waves can be represented as a linear combination of individual sinusoidal waveforms with characteristic frequencies and amplitudes. The auditory nerve generates action potentials at fixed phases within the sinusoid through a process known as phase locking. This allows for the generation of a temporal code, where the intervals between action potentials within the auditory nerve may encode the frequency of a given tone [30]. While individual neurons may not generate action potentials rapidly enough to encode frequencies above 4000 Hz, the Volley principle argues that groups of neurons activated at different cycles of the waveform may collectively represent higher frequencies [33]. Psychoacoustic studies provide evidence for both theories, and both strategies are employed in CI sound processing [34,35].

Pitch processing can be further described by the overall temporal structure of sound. Dynamic features of sound, such as rhythm, syllabic timing, and changes in loudness, are described by the temporal envelope, which refers to slow (1–50 Hz) fluctuations in overall sound amplitude. Temporal fine structure refers to fast-oscillating (>100 Hz) waveforms within the temporal envelope, which encode information about pitch, spatial location, and harmonic structure [36]. The temporal envelope is typically sufficient to encode the gross features of speech, including changes in volume, rhythm, and vowel–consonant boundaries. Accurate perception of temporal fine structure relies on high-frequency hearing; it is critical for music appreciation, sound localization, and understanding tonal speech. The perception of temporal fine structure is often compromised in presbycusis (i.e., age-related hearing loss) and with the introduction of environmental noise, as this creates competing auditory sources [37].

## 4. Cochlear Implant Design and Technical Limitations

CIs are implantable neural prostheses that bypass dysfunctional hair cells within the cochlea to directly stimulate the auditory nerve. As opposed to conventional hearing aids, which amplify sound, CIs convert sound waves into electrical signals, which are processed by the brain as higher-order auditory information.

CIs consist of three main components: a processor, a receiver–simulator, and an electrode array [38]. The external processor captures sound via a microphone and converts key acoustic features into a digital signal. These signals are transmitted via radiofrequency waves to the subcutaneously implanted receiver–stimulator. The receiver–stimulator converts this signal into a series of electrical signals distributed across an electrode array. The electrode array, which is inserted into the scala tympani of the cochlea, distributes electrical signals across multiple contacts to tonotopically stimulate the auditory nerve [17].

In the United States of America, CIs are currently produced by three FDA-approved manufacturers, i.e., MED-EL, Advanced Bionics Corporation, and Cochlear Corporation, which have each shown comparable device performance [39]. CI design may vary by electrode shape, placement, and count. Straight electrodes are typically positioned along the lateral wall of the scala tympani, which can be effective for some cochlear shapes and have been recently modified into shorter and atraumatic designs that are conducive to hybrid stimulation and better preservation of residual hearing [40]. Perimodiolar electrodes curve along the central axis of the cochlea and, therefore, can activate spiral ganglion neurons with less electrical current than lateral wall arrays. The electrode count can range between 16 and 22 electrodes. A higher electrode count can divide the cochlea into finer frequency bands for improved pitch discrimination, but overlapping stimulation between adjacent leads may create interference [41,42]. Finally, hybrid electrodes can provide both acoustic amplification and electrical stimulation to patients with residual hearing. Considerations for CI selection include baseline characteristics, anatomic factors, and long-term goals, which can vary widely between patients (Table 1).

There is also variation in anatomic and surgical considerations. The average cochlear duct ranges from 25 to 35 mm, with a mean width of 6 mm, which has implications for the ideal length of implanted electrodes to avoid cochlear trauma [43]. Across individuals, the cochlea frequently varies in the number of turns, basal and apical width, dimensions of the scala tympani and scala vestibuli, and width of the modiolus—all of which may influence the proximity of electrode contacts to spiral ganglion neurons. A subset of patients with cochlear malformations, such as incomplete partition type 1 or 2, require additional considerations [44,45]. Despite significant anatomic variation between patients, the use of intra- and post-operative imaging or electrophysiological monitoring during cochlear implantation is nonstandard across surgeons, and a “one-size-fits-all” approach is frequently implemented [46]. Thus, there may be optimal electrode selection or placement strategies that are not fully captured by current surgical practices.

Finally, there is significant variation in CI tuning, which may lead to different sound perception outcomes in different users. The processor may employ various strategies to filter sound. For instance, the continuous interleaved sampling (CIS) approach extracts the slow-amplitude temporal envelope and discards the temporal fine structure to optimize speech recognition at the expense of pitch and timbre [47]. While this strategy is employed by most CIs, others have begun to employ strategies, such as fine structure processing (FSP), to preserve some low-frequency phase information for enhanced pitch perception [48].

The process of assigning frequency bands to each electrode also varies between users. Each contact within the CI electrode array captures a frequency band along 200 to 8500 Hz. This range is narrower than a normal-hearing ear, which can perceive around 50 to 15,000 Hz [49]. Since there are significantly fewer electrodes than hair cells, each electrode must capture a broader range of frequencies (hundreds to thousands of Hz) compared to individual hair cells (around 20 Hz) [50]. The assigned frequency bands and location of the electrodes must then accurately recapitulate the tonotopic organization of the Organ of Corti. These assignments are typically calculated using an average cochlea with a fixed duct length and frequency distribution [51]. Thus, there is potential for pitch–place mismatch, where an electrode is incorrectly assigned to a frequency band that is not consistent with cochlear tonotopic organization, particularly when the anatomy of a patient’s cochlea deviates from the average measurements [52,53,54]. Some listeners can adapt over time to these spectral shifts, while other users show partial or limited adaptation [55,56]. Finally, there is a loss of spectral–temporal features through compression strategies that reduce the dynamic range and perceived “contrast” between loud and soft sounds. A comparison of CI sound processing and normal hearing is summarized in Table 2.

## 5. Music Perception in Cochlear Implant Users

Differences in anatomy, surgical practice, and CI tuning have implications for hearing outcomes in CI users. While speech discrimination outcomes in quiet environments are highly favorable in CI users, music perception has been largely underexplored [57]. In this section, we summarize the emerging literature on music perception in CI users.

On objective measures of music perception, such as pitch discrimination, timbre perception, and melodic recognition, CI recipients generally show poorer discrimination compared to normal hearing listeners (NHLs). While no standardized clinical test exists to assess music perception in CI users, several instruments have been developed in the research setting [58]. The Clinical Appreciation of Music Perception (CAMP) test assesses pitch discrimination (identifying the higher note in a pair), timbre perception (identifying the instrument), and melody recognition (identifying a song without rhythm cues). The CAMP test was validated in a cohort of 48 CI users and 10 NHLs and found lower mean melody and timbre recognition scores in CI users compared to NHLs (25% versus 87%, 45% versus 94%, respectively). CI users also showed lower pitch discrimination, with a mean just-noticeable difference of 3.0 semitones compared to 1.0 in NHLs [59]. Other tests, such as the Appreciation of Music in Cochlear Implantees (AMICI), Musical Sounds in Cochlear Implants (MuSIC), and the Montreal Battery of Evaluation of Amusia (MBEA) instruments, include assessments for genre identification, chord discrimination, and identification of music versus noise [60,61,62]. Small cohort studies with these tests demonstrate lower pitch and timbre discrimination in CI users compared to NHLs, but good proficiency with basic rhythm-based tasks [63,64,65]. Some studies suggest that prior musical training and music retraining post-implantation may improve pitch perception in CI users, but outcomes are overall lower compared to NHLs [66].

One of the difficulties in perceiving music accurately is that there is often a mismatch between the frequencies captured by CI electrodes and the natural tonotopic organization of the cochlea. A recent study examined 39 CI recipients with normal cochlear anatomy implanted with the same size electrode and compared the patients’ clinical pitch–place maps to actual place–pitch maps based on the electrode location on flat-panel computed tomographic (FPCT) imaging. The mean frequency-to-pitch mismatch was 1.124 octaves, ranging from 1 to 1.5 octaves across CI users [63]. A mismatch of 1.124 octaves has a significant impact on music perception. Pitch perception is further exacerbated by the challenge of encoding a full frequency range of up to 20,000 Hz using only a limited number of channels (typically 12–22). As a result, CI users were less accurate at differentiating out-of-tune intervals from in-tune intervals. When presented with recordings of “Happy Birthday” with variable intervals between notes, CI users were less able to indicate which recordings were out-of-tune or in-tune compared to NHLs. CI users with single-sided deafness were also asked to listen to the altered and unaltered recordings. These participants were able to indicate out-of-tune and in-tune intervals using the normal hearing ear only, but they were not able to accurately indicate the difference between the intonation of intervals when using the implanted ear [67].

CI users also interpret pitch-related information, such as musical tension, differently from NHLs. Mozart’s Piano Sonata No. 4 in Eb Major, K282, a piece specifically chosen for its unanimity in analyzing tension perceptivity, was played for CI users and NHLs [64]. The melody was then edited to control for different auditory qualities: one randomized pitch at certain notes, one adjusted all notes to be the same volume, one set an equal duration of rest between each tone, and one exaggerated tempo changes. The participants were each asked to rank the tension during each recording using a sliding bar. For the unaltered recording, CI users and NHLs rated tension increased and decreased at similar melodic permutations. Removing tonal cues did not affect the tension ratings of the CI users as much as it did the ratings of the NHLs, but removing loudness cues affected CI tension ratings more than those of NHLs. Removing timing cues affected both the CI and NHL groups [65]. These findings suggest that CI users utilize mainly loudness and tempo cues over pitch to assess the tension of music.

While CI users are less accurate compared to NHLs on metrics such as pitch and timbre, subjective measures of music appreciation vary between CI users. Currently, there is no standardized instrument to assess subjective listening experience. The Music-Related Quality of Life (MuRQoL) instrument was developed in 2017 to assess the impact of music on an individual’s quality of life. The instrument includes a music use questionnaire, which assesses how often and in what ways an individual engages with music, and a quality-of-life questionnaire, which assesses enjoyment and overall satisfaction with music listening. A scoping review identified 11 studies that applied MuRQoL to subpopulations of CI users, including unilateral and bilateral CI users, pre- and post-lingually deafened users, and younger and older adults, and found that reported enjoyment with music listening varies more significantly among CI users than NHLs [68]. Other Likert-scale questionnaires assessing frequency, duration, and enjoyment with music listening have found similarly variable responses amongst CI users, where some report decreased enjoyment with music listening post-implantation, while others enjoy music as much as NHLs [69].

Prior auditory experience appears to play a role in music enjoyment. In a cohort of 23 pediatric CI users, longer duration and better quality of pre-implant hearing were associated with greater music enjoyment post-implantation [63]. In adult users, however, post-lingually deafened CI users were found to report lower music participation and enjoyment compared to pre-lingually deafened users [70,71]. Of the users who report decreased satisfaction, music is often described as dissonant, emotionless, unpleasant, or weak in bass frequencies [72]. The difference in listening experience between pediatric and adult patients is thought to be due to higher levels of neural plasticity in younger patients with prior auditory experience, but the mechanisms are not well understood [73].

The composition of music also impacts CI users’ listening experience. For example, CI users report higher levels of enjoyment when music is simplified to use a smaller set of instruments or filtered to have a lower dynamic range that more closely matches CI mechanics [74]. One publicly available listening rehabilitation program, Angel Sound Training, provides music samples processed to simulate hearing in bilateral CI users. In a cohort of 10 CI users and 25 NHLs, the CI users reported greater enjoyment listening to music processed through the CI-simulation program compared to unprocessed music and even compared to NHLs listening to processed music [75]. Another cohort study found that CI users performed better on an emotional categorization task of musical pieces when the levels of background noise were reduced [76]. The presence of complex sounds that are inaccurately translated by CIs can detract from the overall listening experience.

There are multiple implications of this decreased music perception and music engagement in CI users. Music has demonstrated numerous health-related benefits, including improved memory retrieval in Alzheimer’s disease, reduced severity of anxiety- and depression-related symptoms, reduced anhedonia in schizophrenia, improved pain management, and lessened morbidity associated with loneliness [77,78,79,80]. Music therapy is also associated with lower healthcare-associated costs and risk of harm compared to pharmacologic treatments for similar conditions [81]. Individuals with hearing loss are particularly susceptible to cognitive decline, mood disorders, and social isolation and represent a population that would particularly benefit from music-related therapeutic options [82,83]. Furthermore, the public holds music in high importance both aesthetically and socially, and participation in or enjoyment of music is an essential part of identity, community, connection, and quality of life [84]. Finally, music is considered the richest and most complex acoustic stimulus. Because CI users incompletely perceive the rich spectral features of music, they have still not achieved the goal of neural prosthesis, which is complete sensory restoration.

## 6. Integrating Personalized Medicine into Cochlear Implantation

**Figure 3:**
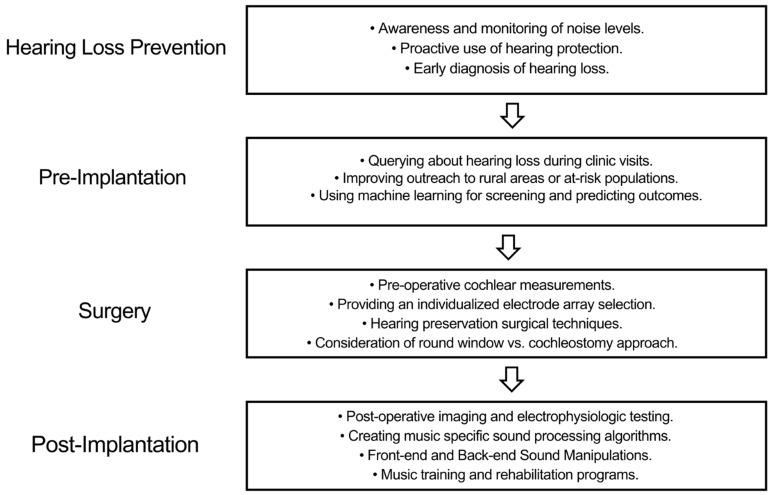
Schematic representing a multi-tiered approach towards integrating personalized medicine into cochlear implantation with the aim of achieving complete sensory restoration.

Given the current situation, we propose a multi-tiered approach towards integrating personalized medicine into cochlear implantation with the aim of achieving complete sensory restoration. Broadly, this approach can be divided into the following stages: prevention, pre-implantation, surgery, and post-implantation (Figure 3), spanning the entire continuum of care. We believe that by both incorporating these personalized medicine approaches and targeting music perception as an outcome measure of complex sound perception, we will inch closer towards complete sensory restoration.

### 6.1. Hearing Loss Prevention

The World Health Organization estimates that approximately 50% of hearing loss in children and adults is preventable [85]. Common causes of hearing loss include age-related changes (35–50%), noise exposure (25–30%), infections, trauma, and exposure to ototoxic medications [5]. As our population continues to age, the incidence of hearing loss and its comorbidities is expected to rise. It will become integral to incorporate hearing loss prevention strategies into workplaces, primary care centers, and personal devices to effectively reach a growing population in need.

One area of research is the prevention of noise-induced hearing loss in recreational settings. While estimates vary, consumer behavior in recreational settings is estimated to comprise up to 30% of noise-induced hearing loss [86]. The National Institute for Occupational Safety and Health (NIOSH) has identified a permissible level of noise exposure to be 8 h of continuous sound at 85 dB, which is comparable to city traffic, a busy restaurant, or a pure tone through average headphones at around 60% volume [87]. Despite established guidelines, most listeners are not aware of their noise exposure levels. Surveys administered on music forums across social media platforms demonstrate that most users would lower their volume if they knew when to do so [88]. Additionally, community efforts to provide hearing protection in high-risk settings, such as at rock and roll concerts, have generally been found to be effective [89]. There are multiple smartphone applications that provide live monitoring of noise levels, including NIOSH Sound Level Meter, Decibel X, and SoundPrint, which also provide crowdsourced maps of noisy and hearing-friendly environments within neighborhoods. Integrating these applications with music and video streaming services may help provide closed-loop feedback to listeners to improve noise exposure habits by raising awareness.

Another important site for hearing loss prevention is in primary care centers or urgent care centers, as this is often where patients with subjective symptoms of hearing loss, ear fullness, or tinnitus first present [90]. Patients are often reluctant to reveal their hearing loss largely due to stigma and the belief that treatment options are limited, and there are no standard guidelines on when to refer patients for hearing loss screening [91,92]. Multiple cross-national surveys have found that up to 50% of primary care providers believe there are few options to manage hearing loss and often do not inquire about symptoms [93,94]. As a result, common screening tests are underutilized in primary care settings [95]. To address this pattern, a recent study of 14,877 patients at 10 family medicine clinics implanted a best-practice Epic Alert to prompt primary care providers to ask, “Do you have difficulty with your hearing?” The study found that referrals for evaluations of hearing loss increased almost fivefold with this prompting and that 93% of audiologist assessments found the referrals to be appropriate [96].

Proactive screening protocols have many benefits. The primary care setting is one of the most accessible clinical sites for early screening, referral, and reinforcing effective treatment, and it may facilitate more eligible patients to receive care. Additionally, addressing hearing loss is an important part of comprehensive health care as adult-onset hearing loss is a modifiable risk factor for dementia, falls, and psychosocial comorbidities. Proactively identifying hearing loss may allow providers to address stigma, establish patients’ goals, and identify hearing restoration options that are in line with individual patients’ wishes.

### 6.2. Pre-Implantation

The early identification of CI candidates is essential for optimizing hearing outcomes, particularly in young patients whose auditory and linguistic systems are still developing. The current CI criteria include individuals with severe to profound sensorineural hearing loss bilaterally or single-sided profound sensorineural hearing loss with limited benefit from binaural amplification. However, only an estimated 21% of individuals who are candidates for hearing aids use them, and about 2–13% of CI candidates have been implanted [97]. This gap highlights the need for improved awareness among referring providers, public health approaches to reach at-risk populations, and systematic screening protocols to ensure timely referrals.

Patient populations at particularly elevated risk include children, patients from lower-income households, and patients in rural areas [98,99]. Children with untreated or incompletely treated hearing loss are at risk of developing language delays, psychosocial complications in school, and social isolation [100,101,102,103]. Adults living in rural areas are at higher risk of absent or delayed treatment for hearing loss compared to patients in urban environments who score similarly on standard hearing loss surveys, such as the Hearing Handicap Inventory for Adults (HHA) [104]. These barriers are largely due to the cost of insurance, geographic barriers to care, and limited public health outreach. Collectively, these limitations suggest that a comprehensive public health approach is necessary to bring CI candidates appropriate treatment, including proactive questioning at primary care or urgent care centers, public health outreach programs targeting schools and rural areas, and on-site social workers or mechanisms to conduct routine social support evaluations at the time of hearing evaluation.

Finally, growing but preliminary data indicate that machine learning algorithms may assist in identifying CI candidates. One study demonstrated that a semi-supervised machine learning model could accurately predict language performance 2 years after cochlear implantation using pre-implant cortical activation patterns on functional magnetic resonance imaging [105]. Another study of 587 patients undergoing CI candidacy evaluation found that a random forest classification model trained on patient demographics, unaided thresholds, and word recognition scores predicted CI candidacy with higher sensitivity, specificity, and accuracy compared to the 60/60 guideline (pure-tone average below 60 dB or word recognition score of less than 60%) [106]. These early studies suggest that there is a potential benefit to a machine-learning-assisted referral guideline to evaluate patients for CI. However, these models must be built on a diverse patient population with data that are routinely collected in a clinical setting, and they must be externally validated [107].

### 6.3. Surgery

Once a patient has been designated as a prime candidate for implantation, there are several new pre-surgical and intra-operative considerations that could be applied to maximize the success of the implantation. As discussed previously, cochlear anatomy can vary widely, and a “one-size-fits-all” approach to cochlear implantation can lead to inaccurate and imprecise pitch perception. Standard CI programming, or default fitting (DF), uses a default frequency allocation across cochlear electrodes based on average cochlear size [108]. Recent advances in precision medicine have influenced the development of a new approach, known as Anatomy-based fitting (ABF), which measures the cochlear duct length in individual patients to select the appropriate electrode type and insertion depth [109]. This approach aims to maximize full coverage of the cochlea, select electrodes that are appropriately sized to the cochlear duct length, and thus reduce frequency–pitch mismatch in patients whose cochlear anatomy may deviate from the average.

One example of ABF is MED-EL’s OTOPLAN software, which uses pre-operative computed tomography (CT) scans to generate a patient-specific map of the cochlea [110]. OTOPLAN software first measures the cochlear duct length on the CT scan. The surgeon then examines the CT scan to input the diameter (largest distance from the round window to the contralateral wall), width (distance between the cochlear walls perpendicular to the diameter), and height of the cochlea. OTOPLAN also calculates the angular insertion depth (AID) to provide surgeons with the best estimate for an ideal electrode array [111]. The ideal AID is 1.5–2 turns, which is approximately 80% of the cochlear coverage [112]. This value balances the risk of cochlear trauma with an electrode array that is too long and the risk of losing base frequencies at the cochlear apex with an electrode array that is too short. Given variations in cochlear size, OTOPLAN software allows surgeons to calculate multiple parameters pre-operatively to optimize CI electrode shape, length, and depth of placement for each patient’s cochlear anatomy.

There are multiple potential advantages to ABF. First, ABF may allow for a deeper electrode insertion depth while avoiding trauma, which can increase the number of electrodes available to encode lower frequencies. Patients with deeper electrode insertion depths report hearing a more complete spectrum of sound, particularly bass instruments, and perform better on music-based metrics because of greater coverage of the cochlea [113]. Second, ABF allows for more adaptable frequency allocation by insertion depth (for instance, extending low-frequency representation for deep insertions and compressing frequency allocation into a smaller cochlear region for shallow insertions). Third, ABF may be more suitable for patients who desire hybrid Cis, as the approach aims to minimize cochlear trauma and preserve functional tissue. Early studies suggest that ABF is associated with better rhythm, pitch, and timbre perception compared to DF [114]. While the data are still preliminary, some small cohort studies report increased enjoyment of music in CI users with ABF compared to those with DF [115,116]. This area is a promising and growing field for improving music perception.

The electrode insertion approach may also influence pitch perception. CIs may be placed via the round window or via a cochleostomy through the basal turn. In the round window approach, the electrodes are inserted through the round window niche and advanced into the cochlea. This approach is generally considered to be minimally traumatic as it does not violate the cochlear otic capsule. Additionally, the self-sealing round window membrane stabilizes the electrode within the scala tympani and reduces migration risk [117]. In the cochleostomy approach, a small hole is drilled into the basal turn of the cochlea, through which the electrode array is placed. Cochleostomies are generally performed when the round window niche is inaccessible or ossified, for inner ear malformations (such as incomplete partition types I or II) and complex or revision cases [118]. Compared to cochleostomies, the round window approach is associated with improved pitch perception post-implantation. This difference is likely because cochleostomies induce direct trauma to cochlear structures, which leads to higher rates of post-operative osseous fibrosis and may affect the impedances of the implanted electrodes [119]. Additionally, the round window approach may place electrodes closer to cochlear neural substrates and decrease the risk of interscalar migration [120,121]. Other considerations include using perimodiolar arrays to reduce spectral interference, as the electrode contacts align more closely with the spiral ganglion neurons within the modiolus, but data on functional impact are still mixed [122,123,124].

### 6.4. Post-Implantation

A precision medicine approach may be incorporated into multiple steps post-implantation, particularly during the aural rehabilitation phase. Electrode activation typically occurs 2–4 weeks after implantation. At this point, the CI is turned on, minimum and maximum current thresholds are calculated for each electrode, and electrode mapping is performed. Jiam et al. demonstrated that using pitch–place maps developed from individual patients’ FPCTs was associated with improved pitch outcomes compared to standard pitch–place maps. This difference was due to an improved ability to assign electrodes to frequency bands that were consistent with individual patients’ cochlear tonotopic organization, which may vary from the frequency distribution of the average cochlea [125]. A more recent randomized control trial in a cohort of 26 CI users also demonstrated improved low-frequency range coverage and melodic recognition following FPCT-based tonotopic fitting [51]. Overall, these studies demonstrate the value of developing patient-specific pitch maps from radiographic information.

There is a range of algorithms used in CI sound processing that may also influence pitch perception. As discussed previously, the CIS approach extracts the slow-amplitude temporal envelope and discards the temporal fine structure to optimize speech recognition at the expense of pitch and timbre. In contrast, the FSP approach preserves some low-frequency phase information within the temporal fine structure to enhance pitch perception. While CIS may be helpful for listening to speech, FSP may be used when listening to music to preserve fine temporal information [48,126]. Other approaches include current steering or simultaneously stimulating multiple electrodes to create additional “virtual” channels and improve frequency resolution, which may reduce spectral interference between channels and may be helpful for polyphonic discrimination or appreciation of timbre [127]. By understanding patients’ priorities, CI sound processing algorithms may be tailored towards individual listening goals.

There are emerging applications for machine learning in refining CI sound processing. A deep-learning-based noise classification algorithm was applied to speech recordings in noisy environments. CI users were asked to listen to the processed recording after inactivating their CI processors’ noise reduction algorithm and then to unprocessed speech using their processors’ original algorithm. CI users were able to detect speech in noisy environments with higher accuracy when using the deep-learning-based algorithm compared to their processors’ original algorithm [128]. Other noise reduction algorithms have shown comparable outcomes [129]. These studies suggest that there may be a role for machine learning algorithms in improving speech perception in noisy environments. Another study demonstrated that a random forest model accurately predicted minimum current thresholds at switch-on and at 6 months after surgery using patients’ evoked compound action potential (ECAP) potentials. This finding supports the use of objective metrics in post-implantation fitting and is especially useful in pediatric patients who may be less able to provide subjective feedback during fitting compared to adults [130]. Overall, machine learning approaches in CI-sound processing and fitting are still in their nascency, with potential to shape sound perception.

Finally, targeting music-related outcomes may also improve pitch perception. There are currently few established guidelines on post-CI auditory rehabilitation, and music rehabilitation is still in its early stages. Most auditory rehabilitation strategies target good speech discrimination, which is necessary to allow CI users to begin navigating the real world. However, the parameters that optimize speech discrimination may not always improve pitch or timbre discrimination, which are aspects of complex sound processing [131]. During electrode activation and tuning, Mapping Law (MAPLaw) values may be adjusted to optimize outcomes. MAPLaw values include minimum and maximum current thresholds and electrode stimulation rates. While data are limited, some studies suggest that higher MAPLaw values may be preferred by CI users during listening, partially due to small improvements in dynamic range [132,133].

There is also a developing interest in the use of music training programs following CI implantation. One such program, Meludia, is a web-based training program endorsed by MED-EL, which includes exercises on discriminating melody, harmony, specialism, and rhythm, as well as excerpts of familiar melodies [134]. CI users who completed a two-month trial of Meludia and audiobook use during rehabilitation demonstrated improvement in pitch discrimination with both Meludia and audiobook training [135]. Other cohort studies demonstrated improvement in musical pattern perception and enjoyment following melody-based rehabilitative training [136,137,138]. Further research is needed to evaluate the efficacy of post-CI music-based rehabilitation programs.

## 7. Conclusions

CIs are highly successful neural prostheses that have allowed people with severe hearing loss to process sound. Over the past 50 years, CI development has made remarkable ground, such that most CI users have adequate speech perception in a silent environment. Despite this great success, there remain significant limitations in CI performance, notably with speech perception in noisy environments and music perception. Much of this discrepancy stems from the fact that present-day CI systems are imprecise and inaccurate, particularly at detecting complex acoustic features, such as pitch and timbre. This discrepancy is a significant limitation. Music perception, a form of complex sound perception, should be used as the gold standard in CI performance assessment as it represents the most complex acoustic stimuli that we confront in real life. In the past decade, there has been increasing interest in improving CI-mediated music perception as it serves as a conduit to restoring normal hearing. This review summarizes multiple potential avenues and open areas for personalized medicine and music-based approaches to bring CI users closer to complete sensory restoration.

## Figures and Tables

**Table 1:** CI-related factors contributing to variable outcomes post-implantation.

Factor	Variability and Impact on Outcomes
CI Manufacturer	MED-EL, Advanced Bionics Corporation, Cochlear Corporation; comparable performance
Electrode Shape	Straight: against lateral wall of scala tympani; include shorter and atraumatic designs for hybrid stimulation Perimodiolar: curve along cochlear axis; may improve pitch discrimination
Electrode Count	Ranges from 16 to 22 electrodes; higher counts allow for finer pitch discrimination but may increase interference
Standard vs. Hybrid Design	Standard: bypass hair cells to directly electrically stimulate the auditory nerve Hybrid: both electric stimulation and acoustic amplification for patients with residual hearing
CI Sound Processing Algorithm	CIS: focuses on temporal envelop to optimize speech outcomes FSP: partially includes temporal fine structure to improve pitch perception
CI Frequency Mapping Method	DF: default frequency allocation across electrodes based on average cochlear size ABF: selects electrode type, insertion depth, and frequency allocation based on each patient’s cochlear duct length
Patient Factors	Pre- or post-lingually deafened, duration and severity of hearing loss, cochlear anatomy, long-term goals

**Table 2:** Comparison of CI sound processing and normal hearing.

Acoustic Feature	Physiology in Functional Cochlea	Function in CI	Implications for CI Users
Frequency range	5 to 15,000 Hz	70 to 8500 Hz	Loss of high-frequency information and difficulty perceiving base pitches
Frequency resolution	3500 inner hair cells, each captures a ~20 Hz frequency band	12–22 electrodes, each captures a frequency band of hundreds to thousands of Hz	Challenges with precise frequency discrimination (e.g., pitch changes such as semitones, speech in noisy environments)
Temporal processing	Detects both temporal envelope and temporal fine structure	Transmits temporal envelope, omits or partially transmits temporal fine structure	Challenges with precise pitch discrimination and timbre perception
Frequency mapping	Tonotopic organization of cochlea (Pitch Placement theory) and temporal code of action potentials in the auditory nerve (Temporal Pitch theory)	Assignment of a frequency band to each electrode based on location along the cochlea DF: uses the average cochlear tonotopic map ABF: tonotopic map developed for each patients’ cochlea	Pitch mismatch (e.g., hearing tones shifted by 1–2 octaves), loss of high or low frequency information
Dynamic range	120-dB dynamic range	20 to 30-dB dynamic range	Less contrast between loud and soft sounds, challenges with emotional prosody, soft speech music dynamics

## Abbreviations

The following abbreviations are used in this manuscript:

CI	cochlear implant
CIS	continuous interleaved sampling
FSP	fine structure processing
DF	default fitting
ABF	Anatomy-based fitting
HHA	Hearing Handicap Inventory for Adults
NIOSH	National Institute for Occupational Safety and Health
AID	angular insertion depth
CT	computed tomography
ECAP	evoked compound action potential
MAPLaw	Mapping Law
